# Toward air-stable multilayer phosphorene thin-films and transistors

**DOI:** 10.1038/srep08989

**Published:** 2015-03-11

**Authors:** Joon-Seok Kim, Yingnan Liu, Weinan Zhu, Seohee Kim, Di Wu, Li Tao, Ananth Dodabalapur, Keji Lai, Deji Akinwande

**Affiliations:** 1 Microelectronics Research Center, Department of Electrical and Computer Engineering, The University of Texas at Austin, Austin, TX. 78758, USA.; 2 Department of Physics, The University of Texas at Austin, Austin, TX. 78712, USA

## Abstract

Few-layer black phosphorus (BP), also known as phosphorene, is poised to be the most attractive graphene analogue owing to its high mobility approaching that of graphene, and its thickness-tunable band gap that can be as large as that of molybdenum disulfide. In essence, phosphorene represents the much sought after high-mobility, large direct band gap two-dimensional layered crystal that is ideal for optoelectronics and flexible devices. However, its instability in air is of paramount concern for practical applications. Here, we demonstrate air-stable BP devices with dielectric and hydrophobic encapsulation. Microscopy, spectroscopy, and transport techniques were employed to elucidate the aging mechanism, which can initiate from the BP surface for bare samples, or edges for samples with thin dielectric coating, highlighting the ineffectiveness of conventional scaled dielectrics. Our months-long studies indicate that a double layer capping of Al_2_O_3_ and hydrophobic fluoropolymer affords BP devices and transistors with indefinite air-stability for the first time, overcoming a critical material challenge for applied research and development.

Bridgman discovered van der Waals bonded black phosphorus crystals in 1914 during high-pressure experiments^1^. While much of its bulk semiconducting properties have been investigated for one hundred years^2^, it is only recently that few-layer black phosphorus or phosphorene, an elemental two-dimensional (2D) layered crystal, has risen to scientific limelight^3^,^4^,^5^. As a physical analogue to the graphite system, black phosphorus has a distinct puckered or buckled structure^2^. Its fully satisfied valence shell results in a direct band gap^2^,^3^, which has been found to scale inversely with thickness, around 1.5–2 eV for a monolayer and 0.3 eV in the bulk limit^4^,^6^. Notably, BP's high mobility (~1000 cm^2^/V·s reported^3^ and ~200 cm^2^/V·s in this study) and sizeable band gap place it at the electronic intersection of graphene, a zero-gap high mobility 2D crystal^7^,^8^, and semiconducting transitional metal dichalcogenides such as MoS_2_, a large-gap low mobility 2D crystal^9^,^10^. In addition, the thickness-tunable direct band gap of BP is ideal for optoelectronics spanning the infrared to visible regimes, with a favorable potential for hyperspectral photodetection^11^,^12^.

A fundamental material challenge for BP films is the lack of air-stability^13^,^14^,^15^,^16^,^17^, a matter of paramount importance for devices that typically operate in ambient conditions. Similar to hygroscopic red phosphorus^1^,^18^, it is found that unprotected black phosphorus can absorb moisture upon exposure to air, resulting in compositional and physical changes of the material with consequent degradation of its electronic properties. This poses a severe problem for prospective device applications in semiconductor technology and flexible electronics^7^,^8^,^9^,^19^,^20^,^21^,^22^,^23^. To date, active research efforts continue to take place to obtain a complete understanding of the degradation process and a pathway towards air-stable BP devices^13^,^14^,^16^,^17^. A number of recent studies have implemented dielectric capping layers to provide stability for material and device research^16^,^24^,^25^,^26^,^27^,^28^.

In this light, we have systematically investigated the stability of unprotected and capped black phosphorus flakes mechanically exfoliated onto insulating substrates. In our study, BP experienced physical degradation in a matter of hours in air, as determined by atomic force microscopy (AFM) and optical imaging, and complete device failure in a few days. Microwave impedance microscopy with nanoscale spatial resolution revealed that samples with thin capping layers, though more air-stable, began to degrade from the edges inward within a few days, a unique mechanism different from previously reported surface degradation^13^,^17^. Intriguingly, the degradation was primarily electronic with only minor changes in flake thickness and volume, in sharp contrast to unprotected samples. Our results indicate for the first time that visual or topographical techniques are generally inadequate for monitoring the air-degradation in capped BP. Moreover, statistics from BP field-effect transistors (FETs) have shown that double layer capping with dielectric and fluoropolymer films afford robust months-long air-stability, suggesting indefinite long-term stability beyond the timescale of hours and weeks reported in previous studies^13^,^14^,^16^,^17^,^28^. The effectiveness of the double layer capping is attributed to the fluoropolymer's hydrophobicity, which prevents moisture from being adsorbed and diffusing to the black phosphorus interface. The simplicity of the capping methods represents a facile route for achieving air-stable black phosphorus or phosphorene devices that can enable basic studies and potential applications.

## Results

**Figure 1:**
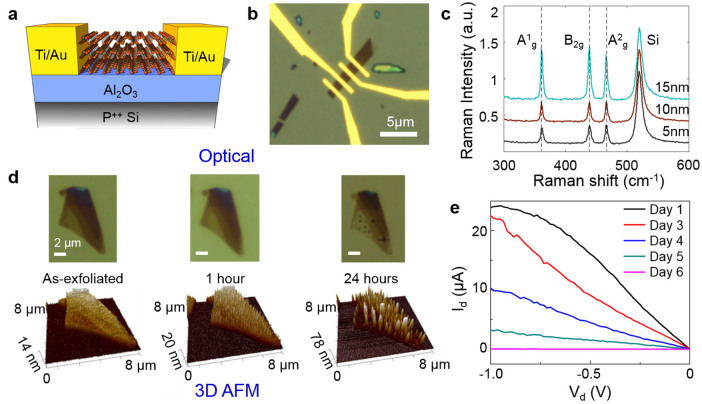
Characterization of bare black phosphorus. (a) Schematic of back-gated BP FET (not to scale). (b) Optical picture of a BP FET device. 25 nm Al_2_O_3_ and 2/70 nm Ti/Au were used as the gate dielectric and electrical contacts, respectively. (c) Typical Raman spectra of BP flakes with thicknesses of 5, 10 and 15 nm. (d) Optical microscope images (top row) and AFM images (bottom row) illustrating the physical degradation of an exfoliated sample in ambient. (e) I_d_-V_d_ characteristic of an uncapped BP device, showing severe degradation of electrical conduction over time.

Multilayer BP samples were exfoliated onto 25 nm thick Al_2_O_3_ on Si substrates. Flakes of thicknesses between 5 and 25 nm were selected by optical and atomic force microscopy. For back-gated FETs, device patterning was done by electron-beam lithography and electrical contacts were formed by electron-beam evaporation of 2/70 nm Ti/Au (Fig. 1a). The optical image of a typical BP device with contact electrodes is shown in Fig. 1b. Raman spectra of typical exfoliated BP flakes showed the three characteristic vibrational modes^4^ (Fig. 1c).

### Exposed black phosphorus

Uncapped BP samples under the normal lighted in-door ambient conditions (temperature 24–27°C, relative humidity 40–45%) experienced material degradation in a matter of hours, which can be seen in the optical and AFM images of a representative flake with initially smooth terraces of different thicknesses in Fig. 1d. This observation of short term degradation of BP upon exposure to air has been also reported in recent studies^13^,^14^,^15^,^16^,^17^. Additional AFM data collected within 24 hrs indicated moisture accumulation on the surface, which resulted in substantial local volume expansion and a cleated surface with sharp spikes. A video clip showing AFM data acquired over one week is available in Supplementary note 10. Corresponding optical images showed increasing optical transparency during the process, indicative of continuous thinning of the flake. As the surrounding Al_2_O_3_ surface area showed no discernible moisture adsorption, we conclude that the degradation is localized to the black phosphorus flake. Electrical measurements on a similar flake showed substantial degradation over two days and complete device failure in a week (Fig. 1e), with the flake vanishing from the substrate surface (see Supplementary Note 1).

The degradation and swelling of uncapped BP is considered to be a consequence of moisture absorption, an attribute of hygroscopic materials^29^. The absorbed moisture has two adverse effects on BP films: i) physical changes such as volume expansion and cleated surfaces (Fig. 1d), and ii) chemical changes towards a liquid phase, which eventually vanishes from the surface (Supplementary Note 1). While the precise composition and stoichiometry of the liquid phase is unknown, energy dispersive x-ray spectroscopy (EDAX) indicates that the droplet-like cleats or swells are oxygen rich (Supplementary Note 2), which may suggest phosphorus oxides in line with recent studies^16^,^17^,^30^, or phosphorus oxyacids as suggested in the early work of Bridgman^1^. Moreover, Raman spectrum of a degraded flake (Supplementary Note 3) displayed peaks characteristic of P-O stretching modes^16^,^31^,^32^,^33^,^34^, lending further experimental support to the suggested oxides or oxyacids. The vast diversity of phosphorus compounds^35^, owing to its wide range of oxidation states (+1 to +5) and coordination numbers, and the possible influence of native impurities or defects in the BP crystal warrant rigorous chemistry research beyond the scope of this work to precisely characterize the air-degraded products.

### Thin-cap black phosphorus

**Figure 2:**
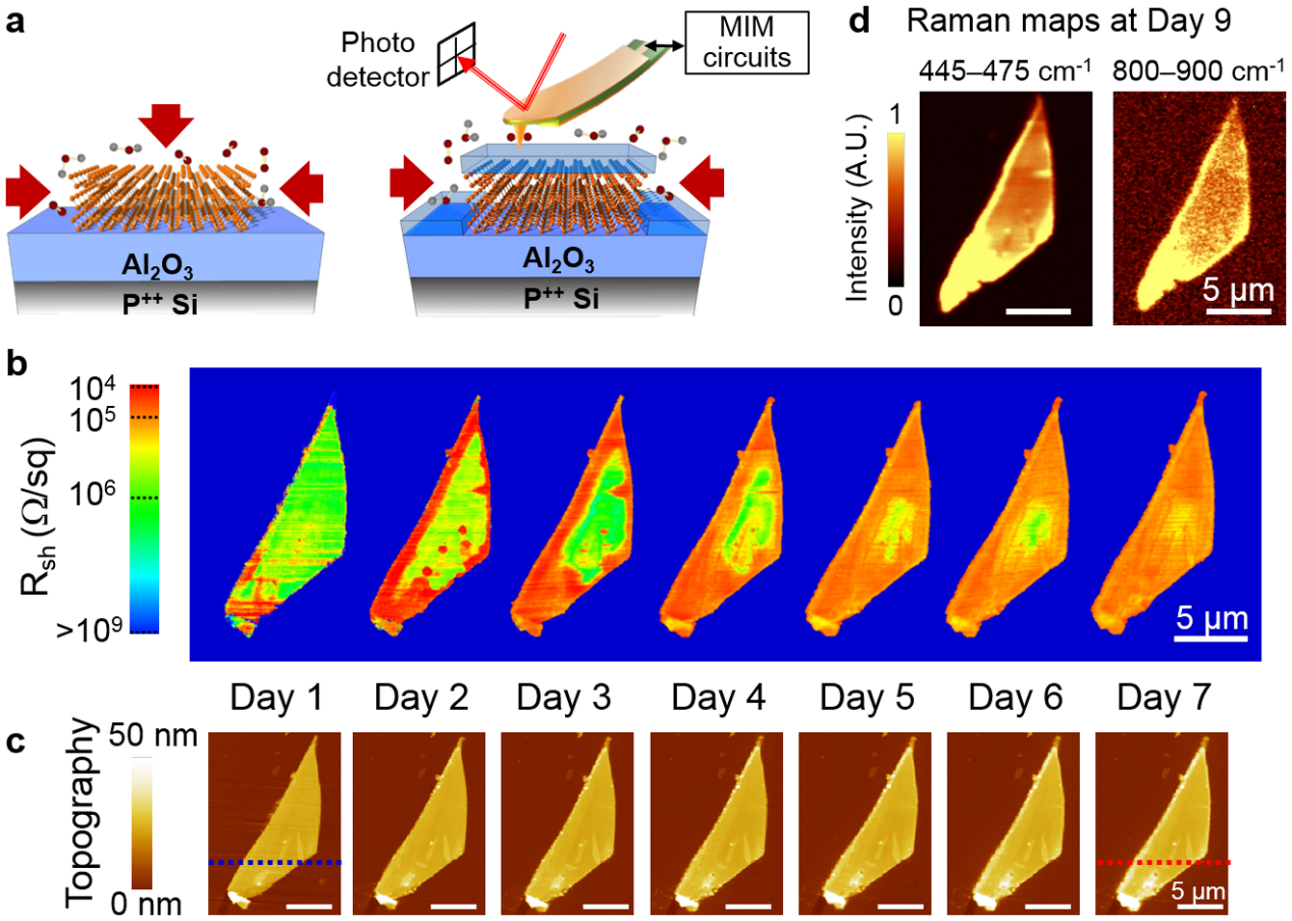
Spatial and temporal evolution of black phosphorus with thin dielectric capping. (a) Schematics of BP samples on Al_2_O_3_/Si substrates without (left) and with (right) a thin Al_2_O_3_ capping layer. Red arrows indicate the possible pathways of oxygen and moisture to react with the sample. The MIM setup is also sketched on top of the thin-cap sample. (b) Local sheet resistance maps derived from the MIM-Real data of a 24 nm-thick flake capped by ~3 nm Al_2_O_3_ layer. The images were acquired daily over one week. Significant conductivity changes initiated from the edges are obvious in the images. The high-conductivity regions in the sample interior (day 2 and 3) are likely due to the local thickness variation. (c) AFM topography of the same flake simultaneously acquired during the same time duration. The line cuts (blue and red dashed lines) in Day 1 and Day 7 are analyzed in Fig. 3d. (d) Raman intensity maps integrated over 445–475 cm^−1^ (A_g_^2^ mode of phosphorene) and 800–900 cm^−1^ (consistent with P-O stretching modes) measured at Day 9. All scale bars are 5 μm.

In order to improve the air-stability of BP films, a thin (~2–3 nm) layer of aluminum was deposited on the exfoliated flakes and oxidized into Al_2_O_3_^25^,^28^,^36^, to serve as a barrier to moisture adsorption, as depicted in Fig. 2a. This range of small dielectric thickness is technologically relevant for scaled 2D FETs^37^. In addition, a thin passivation layer here also entails that the dielectric is thinner than the multilayer BP flakes. To obtain nanoscale electrical information on the thin-cap BP over an extended period of time, an AFM-based microwave impedance microscope (MIM) was employed (Fig. 2a) to simultaneously map out the surface topography and electrical properties with a spatial resolution of 10 ~ 100 nm^38^,^39^. In the MIM experiment, a 1 GHz signal is guided to the tip apex of a customized cantilever probe and the reflected signal is demodulated by the MIM electronics to form local permittivity and conductivity maps of the material. The excitation microwave power is kept low (~10 μW) to avoid non-linear or destructive effects on the sample. Details of the MIM technique are included as Supplementary Note 4. Furthermore, a unique feature of MIM is the near-field capacitive coupling, which enables direct electrical imaging of buried structures with a spatial resolution independent of the free-space wavelength. Compared with other surface-sensitive electrical probes such as conductive AFM or scanning tunneling microscope, the MIM's sub-surface imaging capability is particularly useful for the investigation of capped BP samples in our study, and is generally suitable for the electrical monitoring of spatial or temporal variations in thin films^39^,^40^.

**Figure 3:**
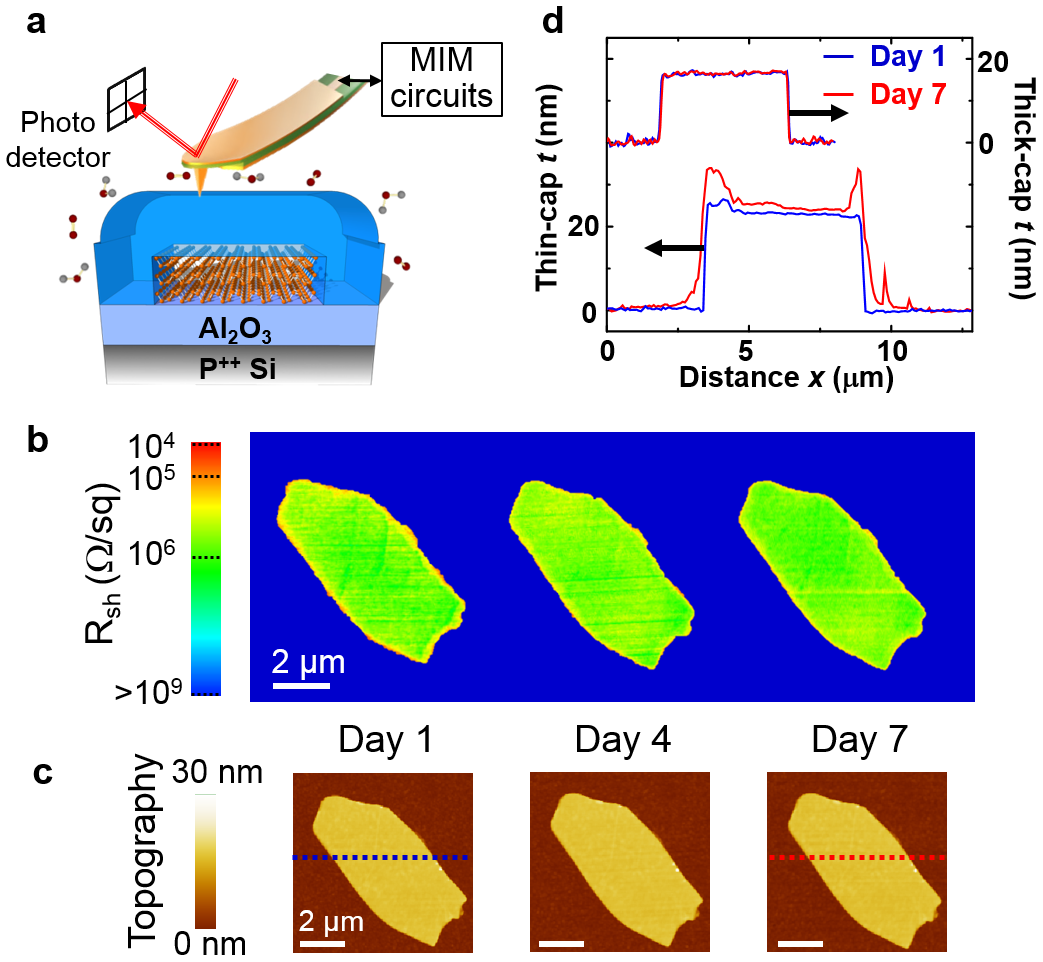
Spatial and temporal evolution of black phosphorus with thick dielectric capping. (a) Schematic of BP sample on Al_2_O_3_/Si substrate with a thick Al_2_O_3_ capping layer against oxygen and moisture in the ambient. The MIM setup is also illustrated. (b) Sheet resistance maps approximated by the MIM-Real data of a 16 nm-thick flake capped by 25 nm Al_2_O_3_. The images were acquired daily over one week. (c) AFM topography of the same flake taken during the same time duration. Neither MIM nor AFM showed discernible changes throughout this period. (d) AFM line profile of thin-cap (bottom) and thick-cap (top) samples. The blue and red lines are associated with Day 1 and Day 7, respectively. Substantial increase in thickness is seen at the edges of the thin-cap sample, while little change is observed for the sample with thick capping. All scale bars are 2 μm.

In order to understand the effect of surface passivation, we measured a BP sample with thin dielectric capping every day over a period of one week by MIM/AFM (raw data shown in Supplementary Note 5). As discussed in Supplementary Note 5, the local sheet resistance, which is displayed in Fig. 2b, can be estimated from the MIM data using numerical analysis^41^. The simultaneously acquired AFM images are shown in Fig. 2c. As seen from the MIM data, the sample right after the dielectric coating was electrically homogenous except for the folded segment at the bottom. The measured sheet resistance R_sh_ ~ 10^6^ Ω/sq is also consistent with our transport data (see below) and reported results at zero gate bias^13^. Strikingly, after one day, the local resistance around the sample perimeter decreased sharply by two orders of magnitude, likely due to doping from the environment, with relatively small change in the surface topography compared to uncapped samples. In other words, unlike uncapped films, the initial degradation of BP with thin dielectric capping is mostly electronic rather than structural. The edge initiated process suggests that a capping layer thinner than the flake may offer poor sidewall coverage (Fig. 2a), hence relatively ineffective against moisture adsorption at the edges. Moreover, degradation initiated from the sample edge dominates the overall degradation process, further suggesting that conformal capping layer with fully covered sidewalls is essential in providing sufficient encapsulation. For longer exposure time in the ambient, the highly conductive regions propagated toward the center of the flake. At the same time, the local sheet resistance at the edges gradually increased. After one week, the entire flake became electrically uniform again (R_sh_ ~ 10^5^ Ω/sq), which is still an order of magnitude more conductive than the initial state. At the same time, the AFM data showed appreciable increase of flake thickness at the edges (see Fig. 3d).

Raman intensity maps collected after the AFM/MIM experiment (at day 9) displayed the three characteristic modes of BP (Fig. 2d and Supplementary Note 6), which corroborates the topographical data regarding the physical preservation of the film. Interestingly, a relatively small and broad bump likely composed of several new Raman modes can be seen in the spectrum at higher wavenumbers (800–900 cm^−1^), which is confined to the BP flake. Compared with published literature^16^,^33^,^34^, this new Raman bump is consistent with the vibrations of mixed phosphorus oxide compounds. Similar new Raman modes were previously observed in doped multi-wall carbon nanotubes as a result of strong dopant-carbon interactions^42^. Based on the AFM/MIM and Raman data, we attribute the degradation of thin-cap BP to partial oxidation initiated from the edges, which drastically impacts the electrical properties while largely preserving the overall physical integrity. With further understanding on their origins, the 800–900 cm^−1^ Raman shifts may become of practical utility in evaluating the purity of BP or phosphorene films similar to the use of the Raman D-mode in characterizing disorder in graphene^43^,^44^.

### Thick-cap black phosphorus

For improved air-stability, a capping layer thicker than the BP flake was deposited on the as-exfoliated samples. As depicted in Fig. 3a, such a thick dielectric coating (~25 nm) is expected to serve as an effective barrier against moisture adsorption at both the surface and edges. Similar thickness effect of an ALD layer was previously used to achieve conformal coatings on pristine carbon nanotubes^45^ and BP^16^,^24^,^25^,^26^,^27^,^28^. The simultaneous topographic and electrical imaging data of a thick capped BP sample are shown in Fig. 3b and 3c (raw data and numerical simulation in Supplementary Note 7). In contrast to the observed electrical inhomogeneity on the thin-cap sample, neither the local sheet resistance nor the surface topography showed discernible changes during the same week-long MIM/AFM monitoring. For a comparison between the two capped samples, selected AFM line profiles at the beginning and the end of the monitoring period are plotted in Fig. 3d. The absence of changes in the MIM/AFM data signifies the preservation of the physical, electrical, and chemical integrity of BP under thick capping over the week-long duration.

### Aging of black phosphorus devices

**Figure 4:**
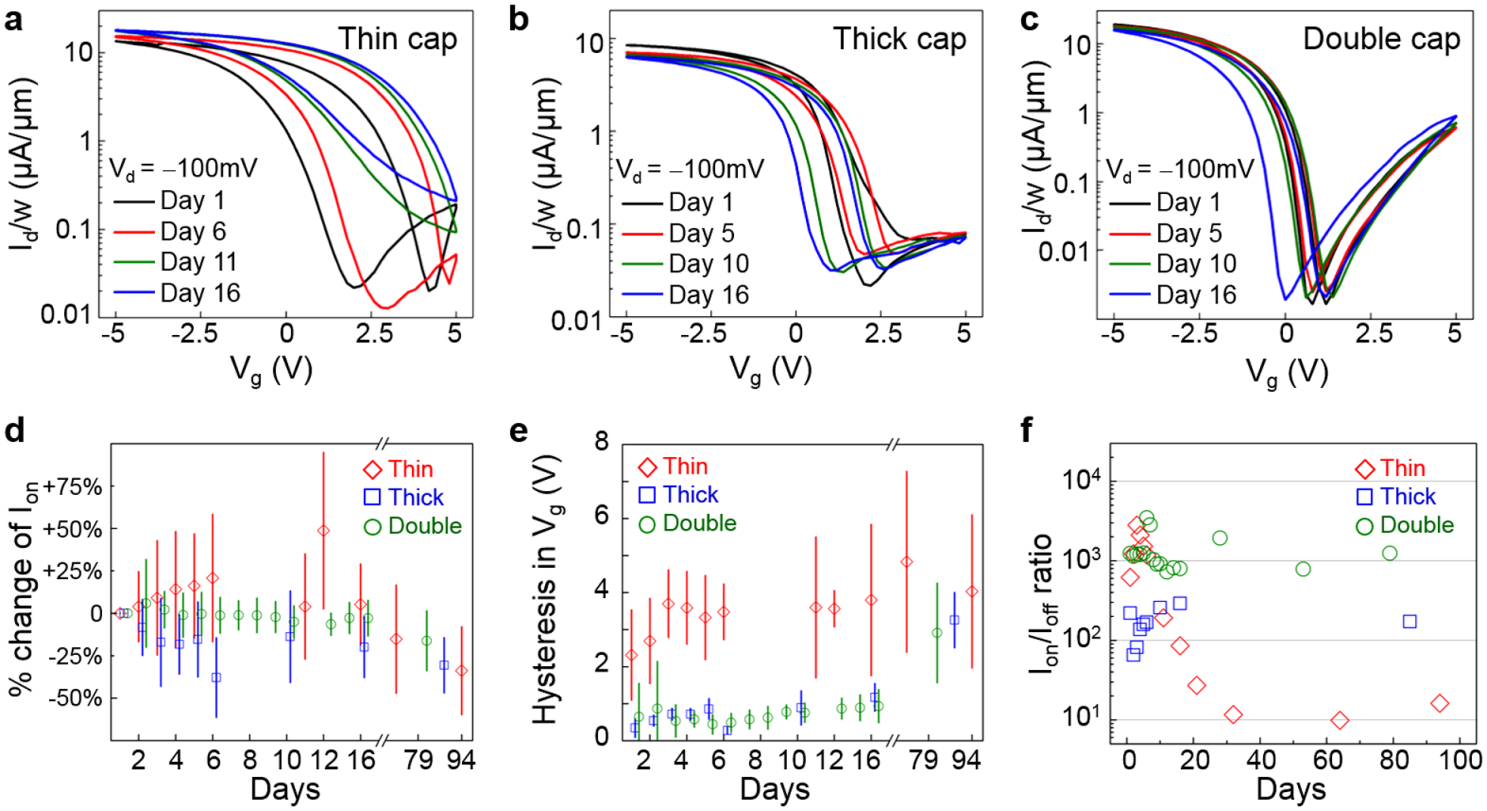
Aging of black phosphorus FETs. (a-c) I_d_-V_g_ plot of BP devices with various capping schemes. The drain voltage V_d_ = −100 mV for all measurements. (a) FETs with thin capping (5 nm ALD-deposited Al_2_O_3_) experienced significant degradation, such as reduced ON/OFF ratio and the increase of gate hysteresis. (b) Thick-cap devices (25 nm ALD Al_2_O_3_) showed less aging effect. (c) Devices with double-layer capping (25 nm ALD Al_2_O_3_ followed by spin-casting of DuPont Teflon-AF fluoropolymer) showed the least aging effect within the same duration. (d–e) Statistical analysis of BP FET devices with different capping methods. The mean values of thin-, thick-, and double-cap devices are presented as red diamonds, blue squares, and green circles, respectively. Vertical bars indicate the range of one standard deviation. (d) The change of ON current (I_on_) with respect to the values at Day 1. (e) Hysteresis in V_g_ between forward and reverse sweeps. For the two performance metrics shown here, the thin-cap FETs exhibited the largest fluctuations and the best stability is obtained in devices with double-layer capping. (f) I_on_/I_off_ ratio of three selected devices. Thin capped device showed sharp degradation, resulting in I_on_/I_off_ ratio of ~10× after a month. In contrast, I_on_/I_off_ ratio of thick capped device was preserved to ~70% compared to Day 1. Double capped device showed the best preservation of I_on_/I_off_ ratio, with negligible change after 79 days. Initial mobility values of the three selected devices are ~200 cm^2^/V-s as shown in Supplementary Note 9.

BP field-effect transistors with different capping schemes were fabricated to elucidate the aging effect at the device level. Electrical characteristics of representative back-gated phosphorene FETs with thin and thick ALD capping are presented in Fig. 4a and 4b, respectively. Detailed sample preparation procedures are described in the Methods section. From Fig. 4a, it is evident that the key device parameters, such as the ON current and ON/OFF current ratio, fluctuated substantially for the thin-cap FET over the course of two weeks. The thick-cap FET, on the other hand, showed better stability over two weeks with the overall ON/OFF switching attributes preserved (Fig. 4b). This increased stability is in agreement with recent studies on passivation of BP with ALD deposited Al_2_O_3_^16^,^28^. However, even thick-capped devices with sidewalls fully covered by the ALD metal oxide dielectric showed some extent of degradation, with the main temporal changes comprising of a negative shift of I_d_-V_g_ transfer characteristics and an increasingly severe hysteresis over time, which may be attributed to the slow diffusion of adsorbates through the Al_2_O_3_ capping layer over an extended time-scale. This indicates that regardless of the thickness, Al_2_O_3_ alone might be inadequate in providing long-term electrically stable BP devices operating in ambient conditions.

To further improve the air-stability of BP transistors, we have developed a double-layer capping scheme by spin-casting a transparent hydrophobic fluoropolymer film (Teflon-AF)^46^,^47^,^48^ on top of the first layer of 25 nm Al_2_O_3_ as described in Supplementary Note 8. The hydrophobicity is crucial for preventing moisture adsorption on the device, resulting in much improved air-stability over the same two-week duration (Fig. 4c). Further insights on the air-stability of phosphorene FETs with thin, thick and double-layer capping schemes were obtained by analyzing the change in electrical performance metrics as shown in Fig. 4d–f. Here, we have collected data on the device performance metrics including ON current, hysteresis in gate voltage, and I_on_/I_off_ ratio over a period of three months for forty-six BP transistors with BP flake thickness ranging from 8.8 to 26 nm. We note that the BP samples used in this study are of good electronic quality with initial mobilities around 200 cm^2^/V·s and comparable to each other at the beginning of the aging experiment (Supplementary Note 9). For thin-cap FETs, severe fluctuations over time and large device-to-device variations are observed, consistent with the strong electrical inhomogeneity observed in the MIM data. I_on_/I_off_ ratio also dramatically decreased by orders of magnitude within a month. The situation is improved in thick-cap samples, which showed relatively moderate variations especially in hysteresis and I_on_/I_off_ ratio. However, ON current level experienced large fluctuation and overall decrease in the thick capped devices. In contrast, devices with double-layer capping feature robust performance metrics with minimal variations over time across devices, which we attribute to the combined benefits of physical and chemical protections against moisture and oxygen molecular species.

## Discussion

The attractive semiconducting properties of reported BP devices with potential mobilities approaching graphene and a direct sizeable band gap similar to monolayer semiconducting transitional metal dichalcogenides have generated substantial interest^3^,^4^,^5^,^12^,^13^. However, the poor air-stability of few-layer BP or phosphorene is a major roadblock that precludes the application of bare materials in normal ambient conditions. In order to take advantage of its attractive properties, a carefully designed capping layer is needed to isolate BP from the environment. While an ultra-thin dielectric coating is desirable for maximum gate control or applications in flexible electronics^19^,^37^,^49^, the present study shows that, without conformal sidewall coverage and moisture resistance from an effective hydrophobic surface, electronic and chemical degradation still occurs and propagates inwards from any exposed edges. This suggests that the aging process of BP may be more complex than originally considered^16^,^17^, with multiple reaction dynamics and pathways contributing to the degradation.

The double-layer coating in this study, which consists of an ALD film followed by the hydrophobic Teflon-AF fluoropolymer, provides a facile route to achieve good air-stability in BP devices. Since direct deposition of fluoropolymers on BP may have adverse effects due to impurity scattering from the solvent residue, the Al_2_O_3_ or other ALD dielectric films are beneficial for ensuring a high quality dielectric-phosphorus interface (Supplementary Note 8). We note that since spin-casted Teflon-AF is a transparent thin film^48^,^50^, its utility as a capping layer is applicable to optoelectronic devices without hindering light-matter interactions.

In conclusion, we have systematically investigated the aging process of multilayer BP by microscopy, spectroscopy, and transport measurements. While bare BP experiences severe physical and electrical degradation within a day, carefully engineered ALD dielectric and hydrophobic polymer capping layers afford much improved air-stability over several weeks without compromising the material properties. The non-invasive MIM, with a unique capability of sub-surface conductivity imaging, was employed to elucidate the aging mechanism of ALD-capped BP. For samples with a thin coating layer, electrical degradation signaled by order-of-magnitude change in local resistivity started from the edges and propagated inward, even though the surface topography is largely preserved. Improved air-stability was demonstrated by capping the flake with a thick Al_2_O_3_ layer and the most air-stable BP devices were achieved with an Al_2_O_3_ coating followed by a hydrophobic fluoropolymer film. Importantly, this work not only provides deep insights on the aging process of BP but also points to a viable route towards robust BP or phosphorene devices, a practical requirement for prospective applications in nanoelectronics, optoelectronics and flexible electronics.

## Methods

### Sample Preparation

BP samples were mechanically exfoliated from bulk black phosphorus (Smart Elements) using conventional scotch-tape method, and transferred onto Al_2_O_3_/Si substrates in ambient cleanroom conditions. The substrate was prepared by depositing 25 nm-thick ALD Al_2_O_3_ on heavily doped p^++^ Si wafer, where the thickness of the dielectric was experimentally selected over 90 nm or 270 nm SiO_2_ substrate to provide better optical contrast of BP flakes with thickness 5–25 nm. Exact thicknesses were measured with Atomic Force Microscope (AFM). The Al_2_O_3_ layer was also used as the back-gate dielectric in electrical measurements. Samples used in MIM experiments were capped after mechanical exfoliation by either the evaporation of ~2–3 nm Al followed by oxidation in 120°C for 20 minutes(thin capping), or ALD deposition of 25 nm Al_2_O_3_ at 250°C (thick capping). Back-gated transistor devices were prepared by patterning resist layer using Carl Zeiss FE NEON 40 SEM system, followed by metal evaporation of 2/70 nm Ti/Au. All devices were designed to have channel length of 1 μm. PMMA EL6 and PMMA A3 were spun at 4000 revolutions per minute (rpm) and baked at 180°C for 2 minutes each in sequence to provide initial capping of BP flakes and to form the resist layer. Thin and thick capping of the transistor devices were formed by ALD deposition of either 5 nm or 25 nm Al_2_O_3_. Double-layer capping was formed by spin-casting DuPont Teflon-AF at 4000 rpm on regular 25 nm ALD Al_2_O_3_ capping layer and cured at 250°C for 30 minutes. The time of exposure to ambient air before applying capping layers was controlled to be less than 1 hours for all samples.

### AFM and optical characterization

Few-layer black phosphorus flakes were exfoliated onto Al_2_O_3_/Si substrates as described above and identified using optical microscope. Flakes with thickness from 5 to 25 nm were targeted in this study. AFM images were collected from a Veeco Dimesion 3100 Atomic Force Microscope under the tapping mode. Non-coated n-doped Si TESP probe was used to achieve sub-nanometer scale AFM scanning resolution. AFM images were captured in ambient conditions. In between AFM scans, the monitored sample was kept in ambient environment with in-door lighting. Raman spectra for typical black phosphorus flakes with thickness of 5, 10 and 15 nm were measured using Renishaw Raman system using a green 532 nm laser with fixed polarization angle. Under 100× magnification and with good focus, the laser beam is 1.2 to 1.5 μm in diameter. Raman shift of ~1 cm^−1^ spectrum resolution was achieved using 2400 l/mm gratings.

### MIM aging experiments

Microwave impedance microscopy (MIM) based on contact-mode AFM was employed to measure topography and local electrical properties simultaneously. Regular AFM images were collected simultaneously with laser feedback from an XE-70 Park AFM. The 1 GHz microwave excitation with V_1GHz_ ~ 20 mV was guided through the electrically shielded cantilever (commercially available from PrimeNano Inc.) to the metallic tip with a radius of ~100 nm. The two output channels of MIM correspond to the real and imaginary parts of the local sample admittance, from which the conductivity and/or permittivity of sample can be deduced. Numerical simulation of the MIM signals was performed by the finite-element analysis (FEA) software COMSOL4.4. The temperature and humidity inside the AFM cabinet were around 25°C and 46.7%, respectively. Samples were stored under similar environment during the week-long experiment. More information about the MIM tip can be found from Ref. 39.

### Transistor device measurements

All electrical measurements were done under ambient conditions in a Cascade summit 11000 AP probing station/Agilent 4156C system using 19 μm-radius tungsten tips from Cascade. The sweep range of back-gate bias was either ±3 or ±5 V, and drain voltage was set to −100 mV with source electrode grounded. The drain bias sweep range was from 0 to −3 V, with varying back-gate voltages. Samples were stored at room temperature at atmospheric pressure, 42 ~ 46% humidity and ambient light between each measurements. All measured data was batch-analyzed with Matlab to extract consistent statistic results over diverse devices. On-current values were evaluated at the same overdrive voltage for all devices in order to eliminate current level fluctuations due to changes in the threshold voltages. Hysteresis was calculated from the difference between forward/backward gate sweeps at the maximum g_m_ values.

## Author Contributions

J.-S.K., K.L and D.A. conceived the original ideas of this study. J.-S.K., W.Z. and L.T. performed material analysis and measurements. Y.L., D.W. and K.L. developed the MIM setup, and performed the measurements and analysis. J.-S.K. and W.Z. prepared and performed measurements of transport samples. J.-S.K. conducted statistical analysis of electrical devices. S.K. and A.D. helped with preparation and analysis of devices with fluoropolymers. D.A. and K.L. led the writing of the paper, and all the authors participated in the discussion of results. The whole project was supervised by A.D., K.L. and D.A.

## Supplementary Material

Supplementary Information Supplementary Information

Supplementary Information Supplementary Media
