# Supplementary material for: Toward air-stable multilayer phosphorene thin-films and transistors
Source: Sci Rep. 2015 Mar 11;5:8989. doi: 10.1038/srep08989 (PMC4355728; doi:10.1038/srep08989)
Supplement: Supplementary Information [file srep08989-s1.pdf]

Supplementary Information

Toward Air-Stable Multilayer Phosphorene Thin-Films and Transistors

Joon-Seok Kim*, Yingnan Liu*,Weinan Zhu*, Seohee Kim, Di Wu, Li Tao, Ananth Dodabalapur, Keji Lai, and Deji Akinwande

* These authors contributed equally to this work.

AFFILIATIONS

1. Microelectronics Research Center, Department of Electrical and Computer Engineering, The University of Texas at Austin, Austin, TX, 78758, USA.

Joon-Seok Kim, Weinan Zhu, Seohee Kim, Li Tao, Ananth Dodabalapur, Deji Akinwande

2. Department of Physics, The University of Texas at Austin, Austin, TX, 78712, USA.

Yingnan Liu, Di Wu, Keji Lai

CORRESPONDING AUTHORS

Keji Lai, kejilai@physics.utexas.edu; Deji Akinwande, deji@ece.utexas.edu

Supplementary Note 1

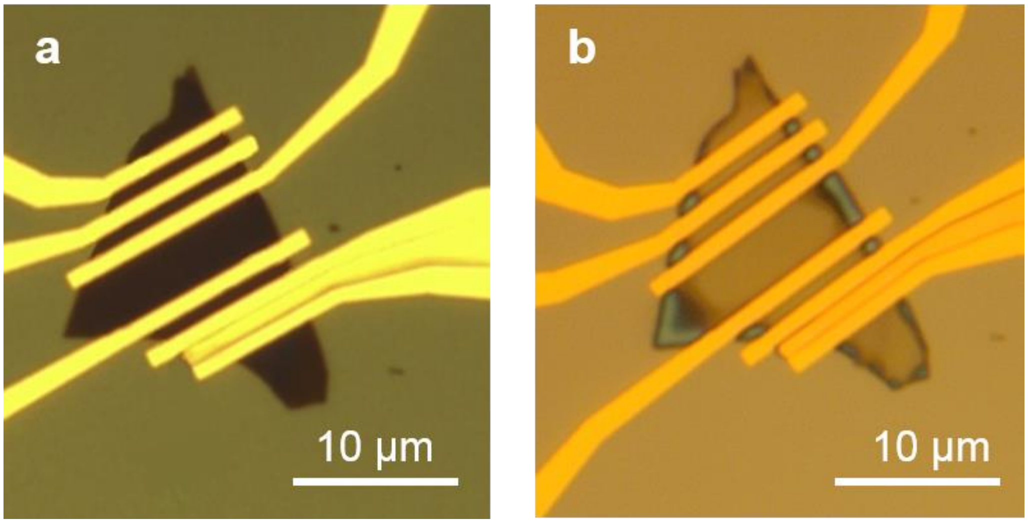

Supplementary Figure 1. Physical degradation of uncapped black phosphorus. Optical pictures of a back-gated bare black phosphorus FET (a) at the time of fabrication and (b) after 7 days in the ambient condition. The black phosphorus sample was fully degraded and disappeared in (b), resulting in complete device failure shown in **Fig. 1e**.

Supplementary Note 2

Compositional analysis of fully degraded black phosphorus was performed by energy dispersive X-ray analysis (EDAX) in the scanning electron microscopy (SEM) chamber. The black phosphorus sample in **Supplementary Fig. 2** was exfoliated onto an Au/Si substrate and kept in air for three days. Before SEM-EDAX analysis, the sample was baked on a hot plate at 100 °C for 10 minutes. From the AFM data, the two droplet-like spots on the black phosphorus surface show a height of 78 nm, much taller than nearby flat regions. The EDAX maps reveal significant oxygen signals on the degraded flake, indicative of oxidation in the aging process.

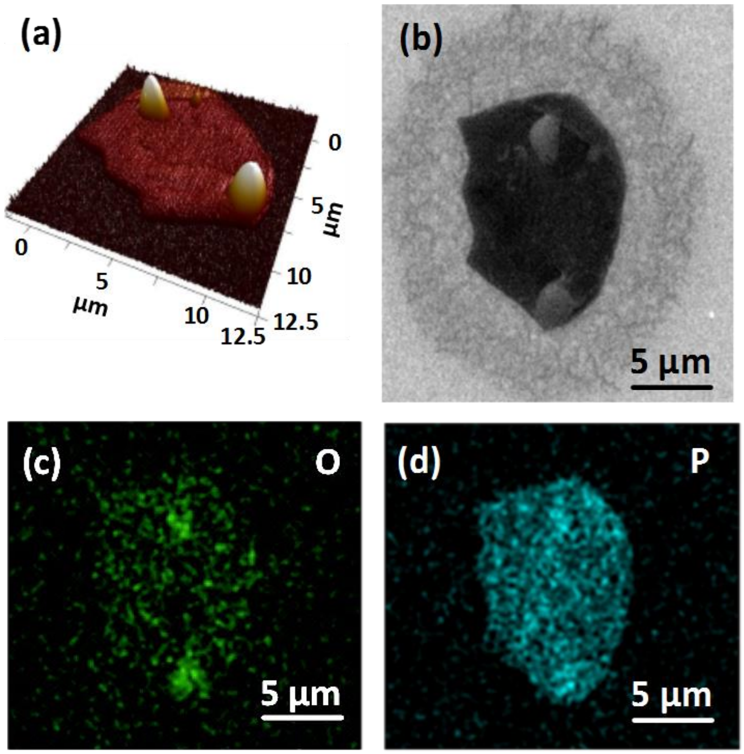

Supplementary Figure 2. EDAX analysis of air degraded black phosphorus. (a) 3D AFM image and (b) SEM image of a degraded black phosphorus sample. (c-d) EDAX maps of oxygen and phosphorus signal in the same sample. The clear oxygen signals indicate certain oxidation process that contributes to the degradation of black phosphorus.

Supplementary Note 3

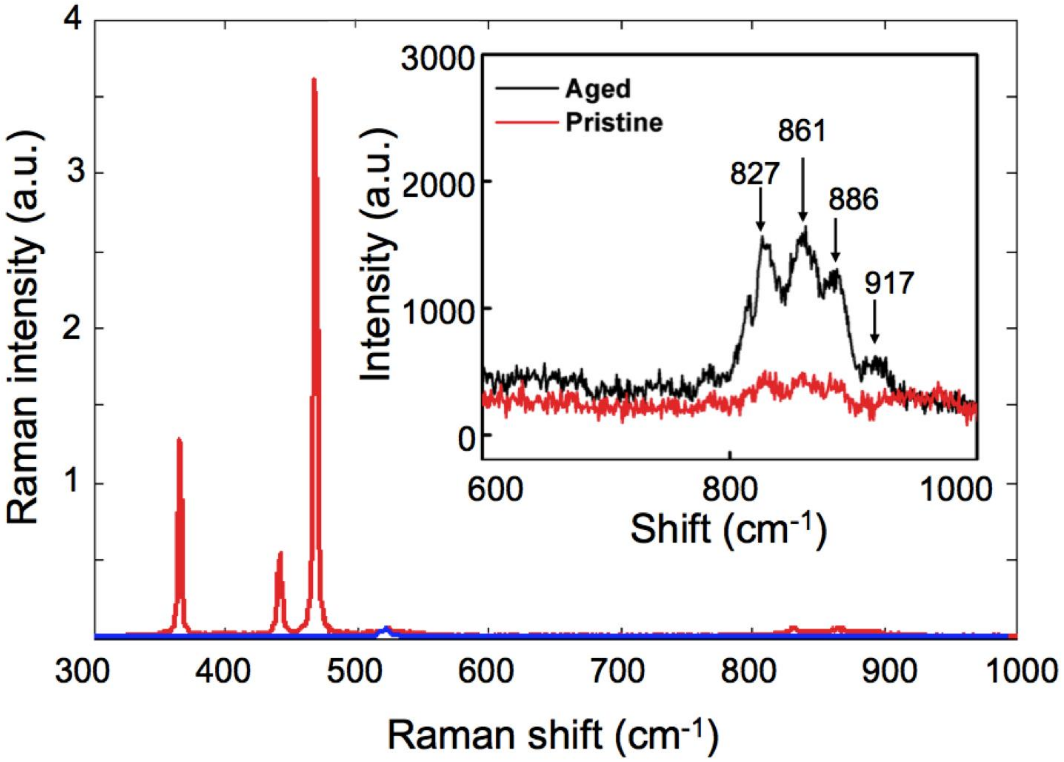

Supplementary Figure 3. Raman spectroscopy of air degraded black phosphorus. Raman spectra of a typical degraded black phosphorus flake (red line) and the Al_2O_3/P^{++} Si background (blue). The inset shows the detailed Raman spectrum of the degraded black phosphorus with several obvious peaks from 800 to 950 cm^{-1} (black line, exposed to air for 3 days) and pristine black phosphorus with much smaller or negligible peaks (red line, within an hour after exfoliation). These Raman signatures are consistent with the reported P-O stretching modes due to oxygen-containing black phosphorus compounds. [S1]-[S5].

Supplementary Note 4

The AFM-based microwave impedance microscopy (MIM) setup is shown in **Supplementary Figure 4**. Details of the shielded cantilever probe are described in [S6]. The 1 GHz excitation signal is guided to the cantilever probe through an impedance-match (Z-match) section [S7]. During the scanning, the near-field interaction modifies the effective tip impedance, which results a change of the reflected microwave. The MIM circuit then amplifies and demodulates this 1 GHz signal. The two orthogonal channels are aligned to the real (MIM-Re) and imaginary (MIM-Im) parts of the tip-sample admittance by adjusting the reference phase to the mixer. Through finite-element analysis (FEA) modeling, the local permittivity (ε) and/or conductivity (σ) of the sample can be deduced from the two MIM outputs [S6]. In general, the MIM-Im signal, which is proportional to the tip-sample capacitance, increases monotonically as a function of the sample conductivity. The MIM-Re signal, which corresponds to loss at 1 GHz, peaks at intermediate conductivities and decreases for both good metals and good insulators.

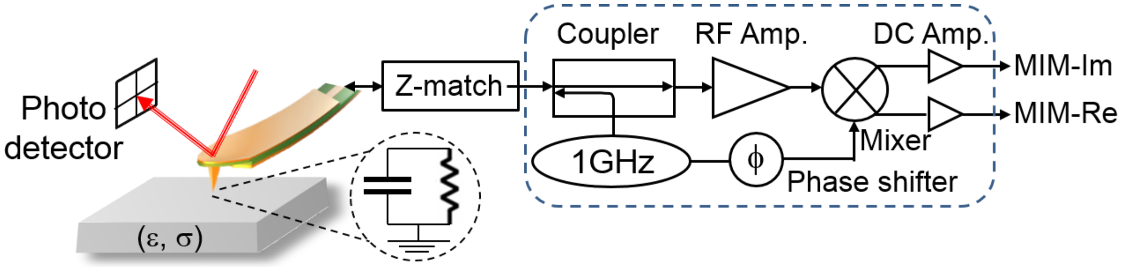

Supplementary Figure 4. Schematic illustration of the MIM setup. The MIM is based a commercial AFM with standard laser feedback for topographic imaging. The tip-sample interaction can be modeled as lumped elements (inset). The MIM electronics shown in the dashed box detect the impedance change and generate the two orthogonal (MIM-Im and MIM-Re) output signals.

Supplementary Note 5

The raw MIM/AFM data of the thin-cap black phosphorus sample in **Fig. 2** are shown in **Supplementary Fig. 5**. The flake with a thickness d = 24 nm was capped by ~3 nm Al_2O_3, which was formed by the deposition of 2 nm Al and the subsequent oxidation at 120 °C. The sample was then measured daily by MIM/AFM over one week. To understand the MIM data in terms of local conductivity, numerical analysis using the commercial software COMSOL4.3 [S6] was performed. The modeling took the sample geometry in **Supplementary Fig. 5d** and computed the real and imaginary parts of the tip-sample admittance (reciprocal of impedance) as a function of the conductivity σ, which is then converted to local sheet resistance $R_{sh} = 1/(\sigma d)$ for comparison with the transport data. Due to the tip wearing from repeated scans, a relatively blunt tip with effective radius of curvature 120 nm was used in the simulation. An electrically uniform black phosphorus was assumed for the 2D axisymmetric model and the result is only semi-quantitative. The MIM-Im data, for example, are strongly affected by topography and non-uniform electrical properties. As a result, we re-plotted the MIM-Re data to approximate the R_{sh} maps in **Fig. 2b**, with its false color scale derived from the simulated response in **Supplementary Fig. 5e**. Note that the MIM-Re signal is a non-monotonic function of R_{sh} [S6] so the insulating substrate is shown as blue in **Fig. 2b**.

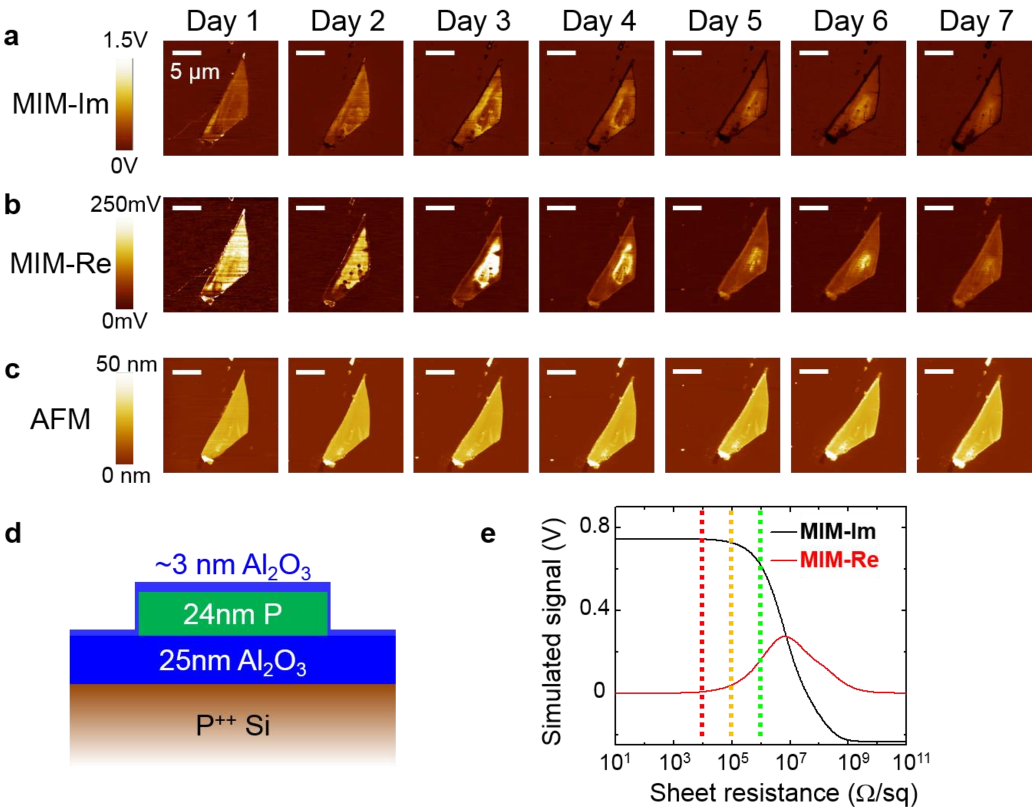

Supplementary Figure 5. MIM/AFM data of thin-cap black phosphorus. (a-c) MIM-Im, MIM-Re, and AFM images of the thin-cap sample acquired over 7 days after the capping. (d) Schematic sample structure. (e) Simulated signals vs. local sheet resistance R_{sh}. The red, yellow, and green lines correspond to $R_{sh}=10^4$, 10^5, and 10^6 Ω/sq,

respectively.

Supplementary Note 6

Raman mapping on the thin-cap black phosphorus sample was performed at Day 9, i.e., after the week-long AFM/MIM experiment. As shown in **Supplementary Figure 6**, all three characteristic peaks (A_g^1, B_{2g}, and A_g^2) were still clearly observed in the Raman maps, indicating the preservation of black phosphorus. The larger signals near the edges are probably due to the thicker material here (see **Fig. 1c**). Interestingly, from **Supplementary Fig. 6d**, significant Raman signals are also seen in the range of 800–900 cm^{-1}. The oxidation process initiated from the sample edges is therefore important for black phosphorus with a thin dielectric capping.

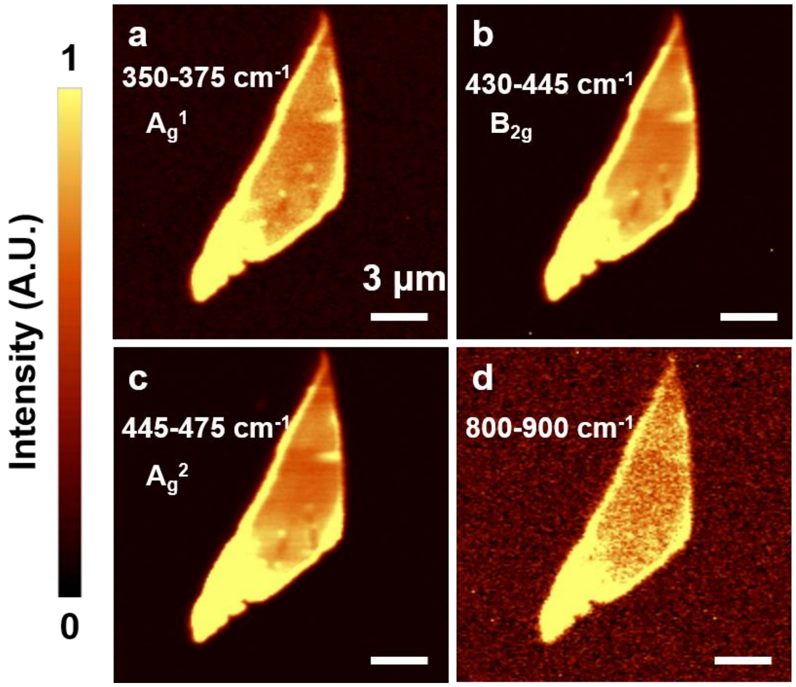

Supplementary Figure 6. Raman mapping of thin-cap black phosphorus. Intensity maps integrating the Raman shift signals within the range of (a) 350–375 cm^{-1} (A_g^1), (b) 430–445 cm^{-1} (B_{2g}), (c) 445–475 cm^{-1} (A_g^2) and (d) 800–900 cm^{-1}. A 488 nm laser was used for Raman measurement.

Supplementary Note 7

A complete set of the raw MIM/AFM data of the thick-cap black phosphorus sample in **Fig. 3** are shown in **Supplementary Figure 7**. The flake with a thickness *d* = 16 nm was capped by 25 nm ALD Al_2O_3. It is obvious from the raw data that neither the local conductivity nor the sample topography was affected by the exposure to ambient oxygen and moisture. Numerical simulation was performed based on the sample geometry in **Supplementary Fig. 7d**. Unlike the thin-cap sample, the thick-cap sample is electrically homogeneous so the local sheet resistance mapping can be obtained by either the MIM-Im or MIM-Re signals. For consistency purposes, we again re-plotted the MIM-Re data to approximate the R_{sh} maps in Fig. 3(b), with its false color scale derived from the simulated response **Supplementary Fig. 7e**. The insulating substrate is again shown as blue in **Fig. 3b**.

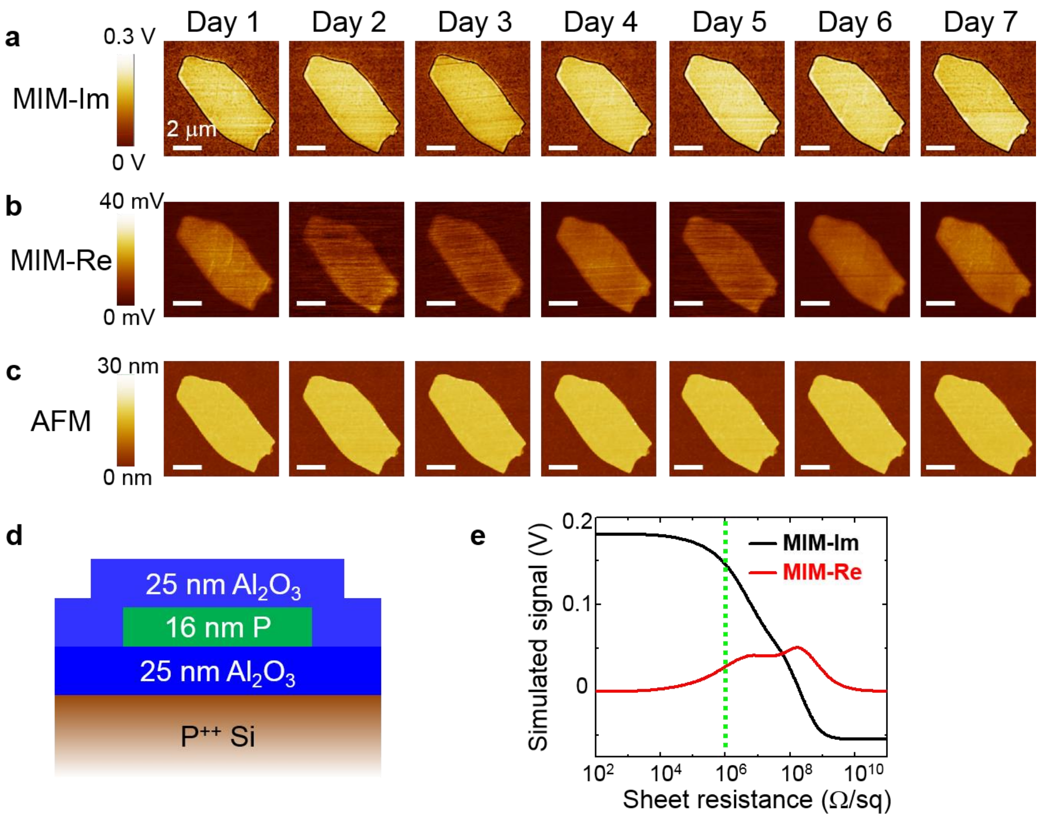

Supplementary Figure 7. MIM/AFM data of thick-cap black phosphorus. (a) (a-c) MIM-Im, MIM-Re, and AFM images of the thick-cap sample acquired over 7 days after the capping. (d) Schematic sample structure. (e) Simulated signals vs. local sheet resistance R_{sh}. The green dashed line corresponds to $R_{sh} = 10^6$ Ω/sq.

Supplementary Note 8

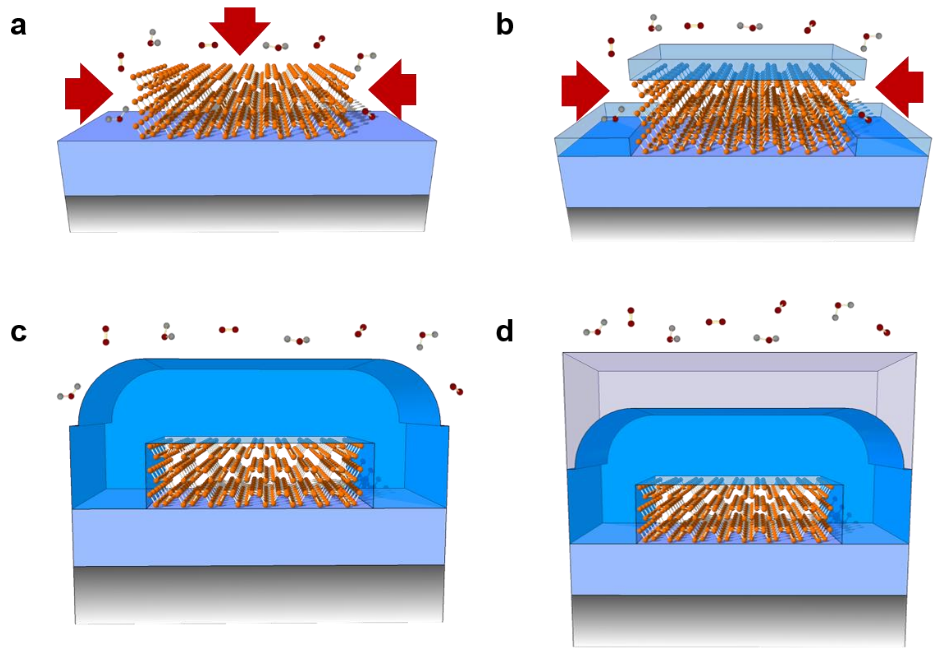

Supplementary Figure 8. Schematics of black phosphorus samples and capping layers. (a) Uncapped black phosphorus, which suffers reaction with the ambient oxygen and moisture from all direction. (b) Black phosphorus with a thin dielectric capping layer that protects the top surface but leaves the sidewalls susceptible to degradation. (c) Black phosphorus with a thick dielectric capping layer with good sidewall coverage. Moisture may, however, slowly diffuse through the capping layer. (d) Black phosphorus with both thick dielectric and hydrophobic fluoropolymer (grey) capping layers, offering the best air-stability in this work. Red arrows indicate dominant path of oxygen and moisture.

Supplementary Note 9

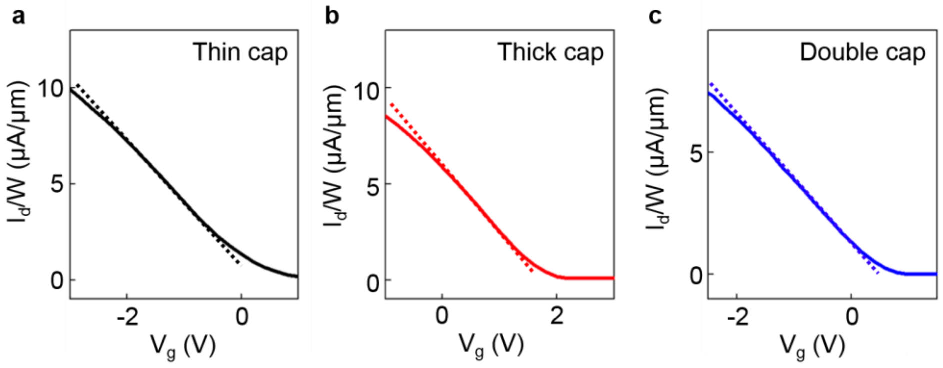

Supplementary Figure 9. Initial mobility of black phosphorus FETs. All FET devices had very decent characteristics and comparable mobilities at the beginning of the aging experiment in Fig. 4. The representative I_d – V_g plots are shown here for typical (a) thin capped (~200 $cm^2/V{\cdot}s$), (b) thick capped (~210 $cm^2/V{\cdot}s$) and (c) double capped (~190 $cm^2/V{\cdot}s$) devices. Dashed lines are linear fits for mobility extraction. Crystal orientation of BP is unknown in this measurement.

Supplementary Note 10

Supplementary Media File. AFM video clip of exposed black phosphorus sample. A black phosphorus flake with initial thickness of 8 nm was monitored by AFM for over one week (170 hours). Local bubbles, a clear sign of degradation and moisture accumulation, reached up to 50 nm height after a week of measurement. AFM measurement was done in ambient condition.

References:

[S1] Rudolph, W. W. Raman-and infrared-spectroscopic investigations of dilute aqueous phosphoric acid solutions. *Dalton Trans.* **39**, 9642-9653 (2010).

[S2] Venkateswaran, C. in *Proc. Indian. Acad. Sci. A.* 25-30 (Indian Academy of Sciences, 1935).

[S3] Carbonnière, P. & Pouchan, C. Vibrational spectra for P4O6 and P4O10 systems: Theoretical study from DFT quartic potential and mixed perturbation-variation method. *Chem. Phys. Lett.* **462**, 169-172, (2008).

[S4] Hanwick, T. J. & Hoffmann, P. O. raman spectra of several compounds containing phosphorus. *J. Chem. Phys.* **19**, 708-711, (1951).

[S5] Wood, J. D. *et al.* Effective Passivation of Exfoliated Black Phosphorus Transistors against Ambient Degradation. *Nano Lett.*, doi:10.1021/nl5032293 (2014).

[S6] Yang, Y. *et al*. Batch-fabricated cantilever probes with electrical shielding for nanoscale dielectric and conductivity imaging. *J. Micromech. Microeng.* **22,** 115040 (2012).

[S7] Lai, K., Kundhikanjana, W., Kelly, M. A. & Shen, Z.-X. Modeling and characterization of a cantilever-based near-field scanning microwave impedance microscope. *Rev. Sci. Instrum.* **79,** 063703 (2008).
